# SH3-Binding Glutamic Acid Rich-Deficiency Augments Apoptosis in Neonatal Rat Cardiomyocytes

**DOI:** 10.3390/ijms222011042

**Published:** 2021-10-13

**Authors:** Anushka Deshpande, Ankush Borlepawar, Alexandra Rosskopf, Derk Frank, Norbert Frey, Ashraf Yusuf Rangrez

**Affiliations:** 1 Department of Internal Medicine III, Cardiology and Angiology, University Medical Center Schleswig-Holstein, Campus Kiel, 24105 Kiel, Germany; anushka.deshpande@uksh.de (A.D.); ankush.borlepawar@gmail.com (A.B.); alexander.rosskopf@uksh.de (A.R.); derk.frank@uksh.de (D.F.); 2 Department of Cardiology, Angiology and Pneumology, University Hospital Heidelberg, 69120 Heidelberg, Germany; norbert.frey@med.uni-heidelberg.de; 3 DZHK (German Centre for Cardiovascular Research), Partner Site Hamburg/Kiel/Lübeck, 24105 Kiel, Germany; 4 DZHK (German Centre for Cardiovascular Research), Partner Site Heidelberg/Mannheim, 69120 Heidelberg, Germany

**Keywords:** SH3BGR, cardiac hypertrophy, SRF signaling, Hippo signaling, apoptosis

## Abstract

Congenital heart disease (CHD) is one of the most common birth defects in humans, present in around 40% of newborns with Down’s syndrome (DS). The SH3 domain-binding glutamic acid-rich (SH3BGR) gene, which maps to the DS region, belongs to a gene family encoding a cluster of small thioredoxin-like proteins sharing SH3 domains. Although its expression is confined to the cardiac and skeletal muscle, the physiological role of SH3BGR in the heart is poorly understood. Interestingly, we observed a significant upregulation of SH3BGR in failing hearts of mice and human patients with hypertrophic cardiomyopathy. Along these lines, the overexpression of SH3BGR exhibited a significant increase in the expression of hypertrophic markers (Nppa and Nppb) and increased cell surface area in neonatal rat ventricular cardiomyocytes (NRVCMs), whereas its knockdown attenuated cellular hypertrophy. Mechanistically, using serum response factor (SRF) response element-driven luciferase assays in the presence or the absence of RhoA or its inhibitor, we found that the pro-hypertrophic effects of SH3BGR are mediated via the RhoA–SRF axis. Furthermore, SH3BGR knockdown resulted in the induction of apoptosis and reduced cell viability in NRVCMs via apoptotic Hippo–YAP signaling. Taking these results together, we here show that SH3BGR is vital for maintaining cytoskeletal integrity and cellular viability in NRVCMs through its modulation of the SRF/YAP signaling pathways.

## 1. Introduction

Trisomy 21, the presence of a supernumerary chromosome 21, results in one of the most common chromosomal abnormalities in humans commonly known as Down’s syndrome, occurring in about 1 in 1000 babies born each year [1]. DS is among the most genetically complex conditions that are surprisingly compatible with human survival [2]. However, congenital heart disease is frequently described in DS patients as the main cause of death in newborns during the first two years of life. The spectrum of CHD patterns in DS has been subjected to variations due to genetic, social and geographical factors that vary worldwide [3]. Thus, DS is one of the commonest disorders with a huge medical and social cost burden and is associated with several phenotypes, including congenital heart defects, leukemia, Alzheimer’s disease, Hirschsprung disease, etc. However, it is still unclear how and why the individuals that are affected by DS display variable phenotypes. Furthermore, the underlying mechanisms of these variable phenotypes are a challenge, making it essential to explore the elementary molecular mechanisms in depth [4].

Gene regulation is one of the mechanisms by which the expression of certain genes is altered, thereby resulting in disease conditions. SRF is one such multifaceted transcription factor that regulates the expression of a variety of genes by binding to the specific promoter sequence CarG box. SRF regulates the expression of a number of heart-specific genes during both embryonic development and pathogenesis [5,6,7]. A small GTPase RhoA is one of the co-inducers of hypertrophic SRF signaling in the heart [8]. The hypertrophic RhoA–SRF signaling pathway has been closely associated with the apoptotic Hippo–Yap pathway in cancer-associated fibroblasts, revealing mutual dependence. Herein, YAP activity is sensitive to SRF-induced contractility and SRF signaling responds to YAP-dependent TGFβ signaling, establishing an indirect crosstalk to control cytoskeletal dynamics [9]. In the heart, the Hippo–YAP pathway is a kinase cascade that inhibits the Yap transcriptional co-factor and controls organ size during development; epicardial-specific deletion of kinases Lats1/2, for example, is lethal at the embryonic level due to the failure in activating fibroblast differentiation, causing mutant embryos to form/undergo defective coronary vasculature remodeling [10]. This evolutionarily and functionally conserved pathway regulates the size and growth of the heart with crucial roles in cell proliferation, apoptosis and differentiation, thus having great potential for therapeutic manipulation to promote organ regeneration [11,12]. In relation with the highly compartmentalized Hippo pathway in cardiomyocytes, during cardiac stress, Mst1 and Lats2 are activated via a K-Ras–Rassf1A-dependent mechanism in mitochondria or through a NF2-dependent mechanism in the nucleus, respectively, where Mst1 stimulates the mitochondrial mechanism of apoptosis by phosphorylating Bcl-xL and Lats2 induces nuclear exit of Yap [13,14,15]. The activation of this canonical Hippo pathway leads to the stimulation of cell death and inhibition of compensatory hypertrophy by inhibition of Yap in cardiomyocytes [16,17].

Despite years of molecular biology-based cardiac research and circulatory understanding, several yet uncharacterized genes are expected to be associated with cardiomyopathies. Towards this end, we performed expressed sequence tags (EST)-based bioinformatic screening of genetic databases of heart and skeletal muscle and discovered several novel genes, one of which is SH3 domain-binding glutamic acid-rich (SH3BGR). It belongs to a gene family composed of SH3BGR, SH3BGRL, SH3BGRL2 and SH3BGRL3, which encode a cluster of small thioredoxin-like proteins and shares a Src homology 3 (SH3) domain (Supplementary Figure S1A) [18,19,20,21,22]. SH3BGR, located in the DS chromosomal region, was first reported by Scartezzini et al. over two decades ago [23] and was, interestingly, later found to be expressed in the earliest stages of mouse heart development [24]. Furthermore, transgenic mice with an FVB (friend leukemia virus B) background overexpressing SH3BGR in the heart did not affect cardiac morphogenesis; however, the fate of these mouse hearts at adult stages is not reported [25]. Thus, we believe that the potential role of SH3BGR in cardiomyocytes is still elusive. We observed significant upregulation of SH3BGR in the hearts of human patients suffering cardiac hypertrophy and a mouse model of heart failure due to transverse aortic constriction, consequently pointing towards its potential involvement in cardiac hypertrophy and associated modalities. Thus, in the current manuscript, we aim at characterizing the molecular functions of SH3BGR using gain- and loss-of-function approaches in neonatal rat ventricular cardiomyocytes.

## 2. Results

### 2.1. SH3BGR Is Confined to Striated Muscle and Upregulated in Cardiac Hypertrophy

**Figure 1:**
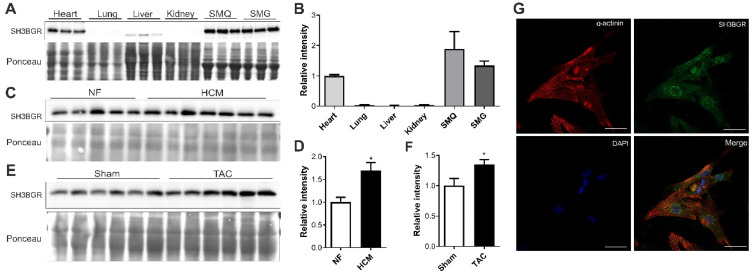
Expression pattern of SH3BGR. SH3BGR expression was observed to be confined to heart and skeletal muscle at protein level from mouse tissue lysates as shown in (**A**); its densitometric analysis is shown in (**B**) (*n* = 3). (**C**) SH3BGR was identified to be upregulated in human patient heart samples suffering from cardiac hypertrophy (HCM, *n* = 7) as compared to non-failing (NF, *n* = 5) samples. Its densitometric analysis is shown in (**D**). SH3BGR upregulation was also observed in mouse hearts subjected to TAC surgery as compared to SHAM as shown in (**E**); its densitometric analysis is shown in (**F**) (*n* = 6). (**G**) Immunofluorescence microscopy suggests sarcomeric and perinuclear localization of SH3BGR in NRVCMs. Statistical calculations were carried out using a two-tailed Student’s *t*-test. *, *p* < 0.05; SMQ, skeletal muscle quadriceps; SMG, skeletal muscle gracilis; TAC, transverse aortic constriction.

SH3BGR was first reported in association with the critical region for Down’s syndrome on chromosome 21 [23,26]. Since then, not much is known about the protein nor its role in cardiac pathophysiology, making it an unusual target to study. In the quest to find a potential role of this protein, we checked its expression in different mouse tissues and found it to be confined to the heart as well as to skeletal muscle, indicating a striated muscle-restricted presence (Figure 1A,B and Supplementary Figure S1B,C). Further, we observed an upregulation of SH3BGR protein levels in the hearts of human patients who suffer from cardiac hypertrophy (as compared to non-failing (NF) human hearts) (Figure 1C,D) and in the mouse hearts suffering cardiac hypertrophy due to biomechanical pressure overload (induced by transverse aortic constriction (TAC)) (Figure 1E,F). Furthermore, endogenous SH3BGR was found to be present at the sarcomere, co-localizing with sarcomeric α-actinin (Figure 1G). Altogether, striated muscle-specific expression, coupled with sarcomeric localization and upregulated protein levels in cardiac hypertrophy, indicates SH3BGR plays an important role in cardiac pathophysiology.

### 2.2. SH3BGR Induces Cellular Hypertrophy in NRVCMs

**Figure 2:**
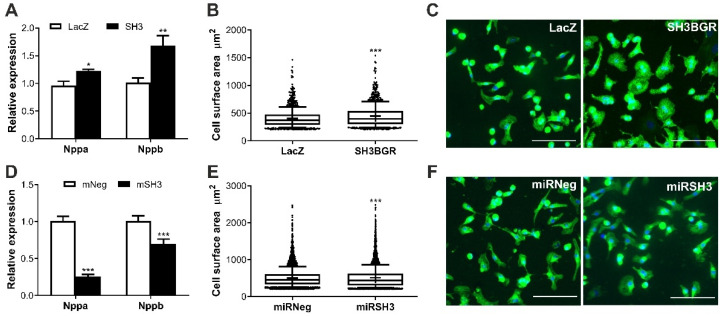
Effect of SH3BGR ablation on hypertrophy in vitro. (**A**) Overexpression of SH3BGR in NRVCMs upregulated fetal genes Nppa and Nppb compared to LacZ control (*n* = 3). (**B**) In line with these results, an increase in cell surface area of NRVCMs was also observed as seen in (**B**); representative images are depicted in (**C**). Contrastingly, on SH3BGR knockdown, this hypertrophic induction was abrogated observed by downregulation of hypertrophic markers (**D**) and reduced cell surface area (**E**,**F**) in miRSH3 condition as compared to miRNeg. Statistical calculations were carried out using the Student’s *t*-test. *, *p* < 0.05; **, *p* < 0.01; ***, *p* < 0.001; SH3, SH3BGR; miRSH3, miRSH3BGR; Nppa, natriuretic peptide A; Nppb, natriuretic peptide B.

To further assess the impact of elevated levels of SH3BGR, which is also observed in DS, we overexpressed SH3BGR in NRVCMs (Supplementary Figure S2A,C); this resulted in the induction of fetal genes, natriuretic peptides A and B (Nppa and Nppb) (Figure 2A) and increased total cell surface area (Figure 2B,C), thereby suggesting that the overexpression is responsible for the induction of hypertrophy in vitro. Contrastingly, SH3BGR knockdown (Supplementary Figure S2B,C) significantly reduced the levels of the hypertrophic markers NppA and NppB, coupled with further reduction in the cell surface area, compared to the control condition (Figure 2D–F).

### 2.3. SH3BGR Regulates RhoA–SRF Signaling in NRVCMs

**Figure 3:**
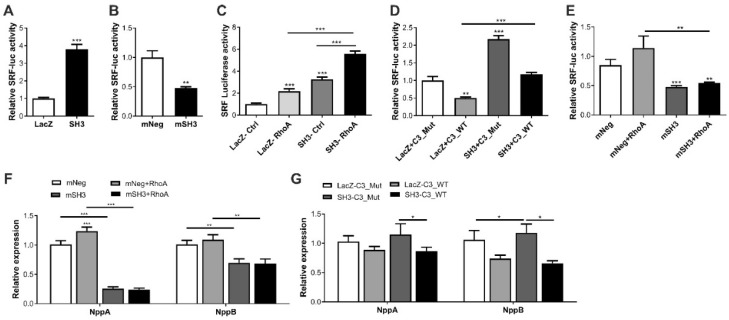
Effect of SH3BGR on SRF signaling-SH3BGR overexpression significantly induced SRF response element-driven luciferase activity (**A**), whereas its knockdown resulted in the inhibition of luciferase activity (**B**). Interestingly, luciferase activity in the presence of RhoA was significantly higher for SH3BGR than LacZ or SH3BGR alone (**C**), whereas the presence of C3 transferase, a RhoA inhibitor, significantly attenuated SH3BGR-mediated luciferase activity (**D**). Similarly, SH3BGR knockdown abrogated the activation of SRF luciferase activity due to RhoA (**E**). These effects were also reflected in the expression of fetal genes, where either knockdown of SH3BGR (**F**) or inhibition of C3 transferase (**G**) reduced the expression levels of Nppa and Nppb. Statistical calculations were carried out using a two-tailed Student’s t-test or two-way ANOVA. *, *p* < 0.05; **, *p* < 0.01; ***, *p* < 0.001; mNeg, miRNeg; mSH3, miRSH3BGR; NppA, Natriuretic peptide A; NppB, Natriuretic peptide B; C3Mut, dominant-negative point mutant C3-E174Q; C3WT, Clostridium botulinum–derived exoenzyme C3-transferase; SRF, Serum response factor.

The serum response factor (SRF) is one of the major transcription factors responsible for cardiomyocyte maturation, structural stability and pathological hypertrophy [8,27]. It plays a significant role in the transcriptional activation of natriuretic peptides and cardiac structural genes that form the core structure of the sarcomere, such as myosin heavy chain 6, 7 (myh 6, 7), myosin light chain 2 (myl2), cardiac alpha actin (ACTC1), etc. Interestingly, in terms of mechanistic relevance of our findings, we explored the Harmonizome, a collection of processed datasets gathered to serve and mine knowledge about genes and proteins, which revealed SRF as one of its transcription factors [28]. Thus, we hypothesized that SH3BGR likely induces cardiomyocyte hypertrophy via SRF signaling in vitro. To test this hypothesis, we studied the effect of SH3BGR overexpression and knockdown on SRF signaling using the SRF-response element-driven firefly luciferase assay/activity. In line with cellular hypertrophy data (Figure 2A–F), we observed a strong induction or inhibition of SRF activity upon SH3BGR overexpression or knockdown, respectively (Figure 3A,B). Interestingly, few of the SH3-domain containing proteins, namely, Tuba, SH3BP1, etc., have earlier been shown to mediate Rho-GTPase signaling, where, RhoA is one of the potent modulators of SRF signaling in the heart [29,30]. Thus, to further dissect the mechanistic insights, we performed a series of luciferase assays in several combinations with overexpression and knockdown of SH3BGR in the presence of RhoA or C3-transferase, a RhoA inhibitor. Our luciferase assay data indicate that the modulation of SRF signaling via SH3BGR is RhoA-mediated, since we observed the synergistic effect of RhoA on the activation of SH3BGR-driven SRF-activity, whereas the presence of C3-transferase abrogated this activation (Figure 3C,D). In contrast, SH3BGR knockdown severely hampered RhoA-mediated induction of SRF signaling (Figure 3E). Similar effects were also observed on the expression levels of the hypertrophic markers Nppa and Nppb (Figure 3F,G). As SH3BGR seems to hamper SRF activity, we investigated its effects on SRF downstream signaling. Moreover, we also observed significant downregulation of several downstream targets of SRF, such as Myh6, Myh7, Myl2, Dystrophin, Actc1 and Acta1, upon SH3BGR knockdown (Supplementary Figure S3A). However, the overexpression of SH3BGR, on the other hand, did not have a significant effect on these SRF target genes (Supplementary Figure S3B). Taken together, our data indicate that SH3BGR induces RhoA-mediated SRF signaling in NRVCMs.

### 2.4. SH3BGR Knockdown Affects NRVCM-Viability and Induces Apoptosis via HIPPO Signaling

**Figure 4:**
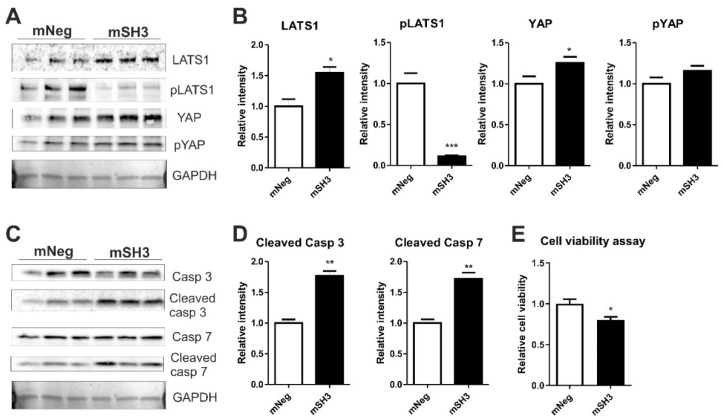
Knockdown of SH3BGR affects cell viability and augments apoptosis via hippo signaling- (**A**) SH3BGR knockdown dramatically reduced the phosphorylation of LATS1 and moderately increased total YAP without altering phosphorylated YAP (pYAP) levels as observed in immunoblots; its densitometric analysis is shown in (**B**). SH3BGR knockdown elevated active, i.e., cleaved, forms of Caspase 3 and 7 (**C**,**D**), indicating increased apoptosis in NRVCMs, which was also reflected in the reduced cell survival determined by cell viability assay (**E**). Statistical calculations were carried out using a two-tailed Student’s t-test. *, *p* < 0.05; **, *p* < 0.01; LATS1, Large tumor suppressor kinase 1; pLATS1, phosphorylated LATS1; YAP, Yes1 Associated Transcriptional Regulator; pYAP, phosphorylated YAP.

**Figure 5:**
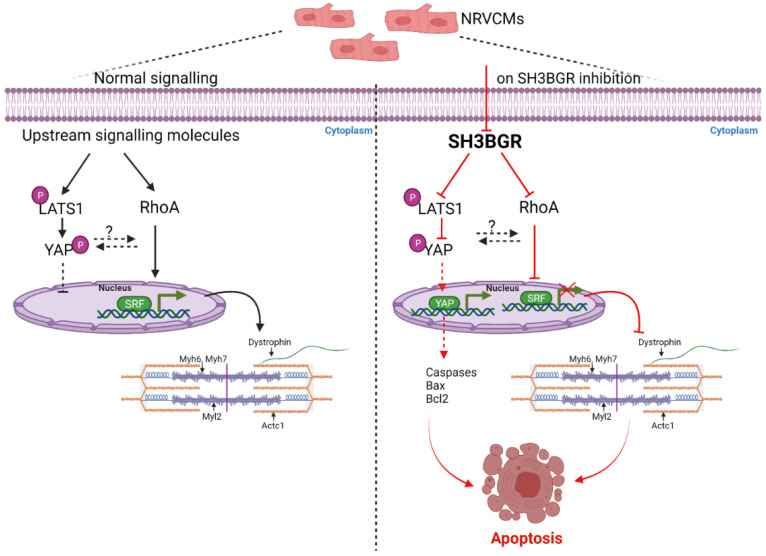
Mechanistic signaling- As observed in the left side panel, LATS1, when phosphorylated, is retained in the cytoplasm and also retains phosphorylated YAP in the cytoplasm, thereby resulting in no transcriptional activation of YAP. Similarly, RhoA activates SRF; the transcriptional activation of SRF is required for genes involved in sarcomere consisting of myosin heavy chain and light chain, actin, etc. On SH3BGR knockdown, as seen in right panel, phosphorylation of YAP is significantly reduced, thereby activating YAP and resulting in probable activation pro-apoptotic genes. Further, the RhoA–SRF axis is inhibited due to SH3BGR inhibition, resulting in hampered SRF activity, thereby resulting in the sarcomeric instability and leading to apoptosis of NRVCMs. SRF, Serum response factor; Myh6, myosin heavy chain 6; Myh7, myosin heavy chain 7; Myl2, Myosin light chain2; Actc1, Actin Alpha Cardiac Muscle 1; Acta1, Actin Alpha 1skeletal muscle; LATS1, Large tumor suppressor kinase 1; pLATS1, phospho Large tumor suppressor kinase 1; YAP, Yes1 Associated Transcriptional Regulator; pYAP, phospho Yes1 Associated Transcriptional Regulator; Bax, BCL2 Associated X; Apoptosis Regulator; Bcl2, B-cell lymphoma 2.

As recent literature postulated SH3BGRL2, a homolog of SH3BGR, to affect the Hippo signaling pathway in renal cell carcinoma, we aimed to find whether SH3BGR affects Hippo signaling in neonatal cardiomyocytes [31]. Intriguingly, SH3BGR knockdown significantly upregulated LATS1 (Large tumor suppressor kinase 1), whereas the levels of its phosphorylated form, i.e., pLATS1, were dramatically reduced (Figure 4A,B). In combination, YAP (Yes1-associated transcriptional regulator) protein levels were strongly increased (Figure 4A,B), suggesting the Hippo pathway to be functionally turned off in the cytoplasm, thereby facilitating the translocation of YAP into the nucleus. This translocated nuclear YAP transcriptionally activates a plethora of pro-apoptotic genes that culminate into the cleavage of executionary caspases-3 and -7 (Figure 4C,D) with expectedly reduced cell viability (Figure 4E). Nevertheless, SH3BGR overexpression did not alter Hippo signaling (except for the moderate increase in total YAP levels) and apoptotic markers (Supplementary Figure S4A–E). Overall, our data suggest that SH3BGR deficiency negatively affects RhoA–SRF and Hippo signaling, resulting in cardiomyocyte hypertrophy and apoptosis, respectively, whereas the overexpression of SH3BGR though robustly activated cellular hypertrophy and SRF signaling, did not affect Hippo signaling and associated cell viability (Figure 5).

## 3. Discussion

First discovered in 1997, SH3BGR is associated with the critical region of Down’s syndrome on human chromosome 21 [32]. Multiple homologs of SH3BGR were soon identified, making it a small family of proteins [33,34]. Since then, limited studies have speculated its roles in the brain, heart and even in carcinogenesis [25,35,36], but the exact function of SH3BGR and its underlying mechanisms remain elusive. Of note, one of the major outcomes of DS is CHD, that affects nearly 1 in 1000 cases of DS worldwide. Although SH3BGR has recently been shown to play a putative role in cardiogenesis as well as heart physiology [24,25], it is still unknown whether SH3BGR is involved in the acquired cardiac dysfunction. Here, using NRVCM as an in vitro study model, we aimed, on one hand to find if increased levels of SH3BGR, as found in Down’s syndrome, affects the cardiomyocyte function. On the other hand, a loss-of-function approach was followed to study the physiological function of SH3BGR.

We observed alterations in SH3BGR levels in the heart of patients with cardiac hypertrophy as well as in the hearts of mouse model of cardiac hypertrophy due to biomechanical stress (TAC). Along these lines, hypertrophic induction and inhibition were observed on SH3BGR via gain- and loss-of-function approaches at in vitro level, respectively, suggesting overexpression, as well as knockdown, has an associated effect against the normal functioning of cells. Digging deeper to understand the mechanistic relevance of these experimental findings, through the Harmonizome database, we identified SH3BGR as one of the transcriptional targets of SRF, a major transcription factor governing the transcription of sarcomeric genes and cardiomyocyte hypertrophy. Interestingly, using a gain-of-function approach, which we believe mimics the Trisomy 21 condition, wherein a gene of interest is overexpressed due to presence of an extra gene copy, we observed that elevated levels of SH3BGR are sufficient for the activation of SRF signaling and cellular hypertrophy in NRVCMs. The loss-of-function approach further strengthened these findings, suggesting a probable mechanism of CHD in DS patients, at least in part. Studies using patient-derived tissue biopsies, induced pluripotent stem cell-derived cardiomyocytes, or in vivo models may be utilized to further validate our findings. Furthermore, we established RhoA to be an intermediary in the activation of SH3BGR-driven SRF signaling, both of which are important in regulating the actin cytoskeleton and sarcomeric homeostasis. The sarcomere is mainly formed of myosin heavy chain and light chain molecules, along with actin. These work in an organized manner for the proper function of contraction and relaxation of beating cells, thereby maintaining cellular homeostasis. Thus, alterations in the levels of any of these proteins could lead to structural and functional instability, which we observed upon SH3BGR deficiency. Thus, we believe that our findings are of direct clinical relevance, particularly in DS patients with CHD, which may be exploited in the future for therapeutic interventions.

Yin et al. [31] have recently provided the first evidence of a homolog of SH3BGR (SH3BGRL2) to regulate the Hippo signaling pathway. In our current study, we found that the knockdown of SH3BGR induces apoptosis via Hippo signaling. Normally, YAP transcriptional activity is shut down on activation of Hippo signaling and vice versa [11]. Being transcriptionally inactive, LATS1 is phosphorylated and, in turn, subsequently phosphorylates YAP, thereby causing YAP retention in the cytoplasm, whereas, on transcriptional activation, YAP is translocated to the nucleus and further triggers downstream genes [11]. Intriguingly, we observed decreased phospho-LATS1 levels and increased YAP levels upon SH3BGR knockdown, indicating YAP to be translocated to the nucleus, which likely resulted in the reduced cell viability and activation of apoptosis via executory caspases 3 and 7. Notwithstanding, though not significant, the overexpression of SH3BGR exerted similar effects on YAP signaling. Although it is not clear from these data why we did not observe contrasting results with SH3BGR overexpression or knockdown on YAP signaling, the plausible reason could be the involvement of yet unidentified player(s) or independent mechanisms, which necessitates further evaluations. Importantly, however, the results from both overexpression and knockdown studies highlight the fact that SH3BGR is essential for normal homeostasis of cardiomyocytes only when present in optimal levels and its either up- or down-regulation is harmful for the cells.

In summary, to the best of our knowledge, this is the first report where the mechanistic insights into how loss- or gain-of SH3BGR differentially affects cardiomyocyte pathophysiology is reported. The overexpression of SH3BGR, which mimics DS condition, significantly activates cardiomyocyte hypertrophy via RhoA/SRF signaling, whereas SH3BGR knockdown abrogates cellular hypertrophy, leading to a combination of sarcomeric dysfunction, activation of apoptosis and reduced cell viability via alterations in the RhoA/SRF and Hippo signaling pathways in cardiomyocytes (Figure 5).

## 4. Materials and Methods

### 4.1. Cloning of SH3BGR Vectors

The expression construct for RhoA was generated as described in Rangrez et al. [8]. The construct for overexpression of SH3BGR was cloned from mouse heart cDNA using primers 5′-GCTGGCACCATG-3′ and 3′-GCTGGGTCGCCCTA-5′ in the pDONR221 gateway cloning vector by two sequential ORF and adaptor PCRs. The cleaned product from the adaptor PCR was then cloned into a donor vector pDONR221 following the manufacturer’s instructions (Thermo Fisher Scientific, Planegg, Germany). Knockdown of SH3BGR in NRVCMs was achieved by cloning the respective synthetic microRNAs using the BLOCK-iT polymerase II miR RNAi Expression vector kit via a two-step reaction culminating the integration of synthetic microRNAs into the Gateway cloning vector pDONR221 (Thermo Fisher Scientific).

Adenoviruses encoding full-length mouse SH3BGR cDNA and synthetic microRNAs were further generated for use in the NRVCMs system using the ViraPower adenoviral kit (Thermo Fisher Scientific) following the manufacturer’s protocol. In brief, previously cloned cDNA or synthetic microRNAs in the pDONR221 vector were transferred into the pAd/CMV/V5-DEST destination vector. The construct was then digested with PacI (10 U/uL; Thermo Fisher Scientific) restriction enzyme and transfected into HEK293A cells to produce the respective adenoviruses. The titration for the adenoviruses was performed by staining virus-infected HEK293A cells with fluorescent anti-Hexon antibody. A β-galactosidase encoding adenovirus (Ad-LacZ; Thermo Fisher Scientific) served as a control for the experimental setup.

### 4.2. Antibodies

The antibodies used for the various experiments in this study were as follows: α-actinin, mouse monoclonal (1:200; Sigma, Germany); α-actinin, rabbit polyclonal (1:400; Abcam, Germany); α-tubulin, mouse monoclonal (1:8000; Sigma); caspase-3, rabbit polyclonal (1:1000; Cell Signaling Technology, Taufkirchen, Germany); cleaved caspase-3, rabbit monoclonal (1:400; Cell Signaling Technology); caspase-7, rabbit polyclonal (1:1000; Cell Signaling Technology); SH3BGR rabbit polyclonal(1:1000; Proteintech, St. Leon-Rot, Germany); LATS1 (1:1000; Cell Signaling Technology); p-LATS1 (1:1000; Cell Signaling Technology); YAP (1:1000; Cell Signaling Technology); p-YAP (1:1000; Cell Signaling Technology).

### 4.3. Isolation of NRVCMs

The cell system used for the experiments in this manuscript is the primary neonatal rat ventricular cardiomyocytes, or NRVCMs. These cells were harvested and prepared for experimental use as described previously [37]. In brief, the left ventricles of 1–2-day old Wistar rat babies (Charles River, Lyon, France) were harvested and chopped in ADS buffer containing 120 mmol/liter NaCl, 20 mmol/liter Hepes, 8 mmol/liter NaH2PO_4_, 6 mmol/liter glucose, 5 mmol/liter KCl and 0.8 mmol/liter MgSO_4_; pH 7.4. For releasing the individual cardiomyocytes from compound chopped tissue mass, between five and six enzymatic digestion steps were performed with 0.6 mg/mL of pancreatin (Sigma) at 37 °C and 0.5 mg/mL of collagenase type II (Worthington, Columbus, OH, USA) in sterile ADS buffer. Subsequently, the compound cell suspension was passed through a particular cell strainer with the final addition of newborn calf serum to stop enzymatic digestion of cell mass. The cardiomyocytes were separated from cardiac fibroblasts using a Percoll gradient (GE Healthcare, Chicago, IL, USA) centrifugation step and were cultured in DMEM with additives such as 10% FCS, 2 mM penicillin/streptomycin and L-glutamine (PAA Laboratories, Pasching, Austria) to support the growth. Adenovirus infection of NRVCMs in DMEM supplemented with penicillin/streptomycin and L-glutamine, but lacking FCS, was performed 24 h post-harvest. The cells were harvested 72 h post-infection.

### 4.4. Co-Localization Analysis of SH3BGR with α-Actinin

The co-localization between SH3BGR and α-actinin was observed in NRVCMs using the LSM800 Zeiss laser-scanning microscope with the help of the ZEN-blue software package. The cells were seeded in a 12-well plate that had a collagen-coated coverslip in each well. Following the adenoviral infection and incubation phase, NRVCMs were first fixed with 4% PFA for 5 min and then, in one step, permeabilized and blocked with 0.1% Triton X-100 in 2.5% BSA in saline (PBS) for 1 h. The cells were then incubated for 1 h with primary antibodies using the following dilutions: polyclonal rabbit anti-SH3BGR (1:200) and monoclonal mouse anti- α-actinin (1:200; Sigma) for co-localization observation. The respective secondary antibodies conjugated to either Alexa Fluor-546 (AF546) or Alexa Fluor-488 (AF488) (Thermo Fisher Scientific) were incubated for 1 h with the same dilution of 1:200 in 2.5% BSA in PBS, along with the nuclear stain DAPI (1:500). FluorSave reagent (Merck Millipore, Burlington, MA, USA) was used as a mounting medium. Fluorescence micrographs were taken using the aforementioned Zeiss LSM800 confocal microscope with a Plan-Apochromat 40/1.4 oil differential interference contrast (UV)-visible IR objective at room temperature. Image pixel size was set to optimal for individual image acquisitions. The pinhole for the acquisition was adjusted to 1 airy unit or less for each laser line. The AF546 and DAPI channels were acquired via GaAsP-Pmt detectors, while the AF488 channel was acquired with a Multialkali-Pmt detector with gain settings between 600 V and 700 V. The laser power for excitation variably ranged from 0.2 to 0.8%.

### 4.5. Immunofluorescence Microscopy for Cell Size Measurement

The cell size measurement of NRVCMs was studied in NRVCMs by immunofluorescence microscopy. NRVCM preparation and staining were performed as described in two separate sections above. Monoclonal mouse anti-α-actinin (1:200; Sigma) was used as the primary antibody for cell size measurements due to its specificity to sarcomeric α-actinin. The respective secondary antibody conjugated to Alexa Fluor-488 (Thermo Fisher Scientific) was incubated for 1 h at a dilution of 1:200 in 2.5% BSA in PBS along with nuclear stain DAPI (1:500). FluorSave reagent (Merck Millipore) was again used as a mounting medium. Fluorescence micrographs (10 per coverslip) were taken with a Keyence fluorescence microscope of series BZ 9000, at X10 objective (Plan Apochromat, NA: 0.45). Images were acquired using the BZ-II image viewer software (Keyence, Oaska, Japan version 2.1) using a built-in camera at room temperature and were processed and analyzed by a BZ-II Analyzer (Keyence, version 2.1) as detailed below.

Cell size was measured using the HybridCellCount software module from Keyence with the fluorescence harvest kept at single-extraction mode. First, the fluorescence intensity thresholds were set for a moderately intense reference picture. Thereafter, α-actinin whole-cell staining was set as the target area and the blue DAPI-stained nuclei were then extracted from each green target area to determine the number of nuclei per target area, i.e., cell. Then, MacroCellCount was performed, applying the settings from the reference picture to each target picture from one set of experiments. Results were manually filtered in MS excel for the following criteria: (a) 200-µm^2^ < target area < 2500 µm^2^ (size filter); (b) extraction from target area = 1 (one nucleus filter, as neonatal cardiomyocytes have only one nucleus); (c) area ratio 1 < 30% (cell surface to nucleus ratio filter, to avoid apoptotic cells). The statistical analyses were performed using GraphPad Prism (version 5). An equal distribution of the data in each group was tested by the Shapiro–Wilk test. The samples were then compared according to the Student’s *t-*test. *n* > 900 (each condition, depending on the cell density).

### 4.6. MTT Assay for Cell Viability

NRVCMs that were cultured in 24-well plates for viability check were infected with RhoA, SH3 and miRSH3 adenoviruses and incubated for 72 h, where miRNeg and LacZ served as controls as per the mentioned conditions. An MTT labeling reagent (in situ cell proliferation kit, MTT I, Roche Applied Science, Penzberg, Germany) was added at a 10% concentration of total DMEM volume to each well. Plates were then incubated for 4 h in a cell incubators’ humidified atmosphere (37 °C, 5% CO_2_). After this incubation phase, an MTT solubilization solution (cell proliferation kit MTT, Roche Applied Science) was added to each well in a quantity 10 times higher than the initially added MTT labeling reagent and was further incubated overnight under the same conditions. After complete solubilization of purple formazan crystals, spectrophotometric absorbance was measured using a 96-microplate reader on an Infinite M200 PRO System (Tecan, Life Science). The percentage of viable cells from each condition was plotted as relative to the negative control. Different groups were then compared according to the Student’s *t-*test. All the experiments were performed in hexaplicate or octuplicate and repeated three times.

### 4.7. RNA Isolation and qRT-PCR

Total RNA was isolated from NRVCMs or human/mouse hearts or other mouse tissue samples using a cell-lysis reagent TriFast (Peqlab, Burladingen, Germany) and a Precellys homogenizer with coarse and fine plastic beads (only for mouse and human samples), following the manufacturer’s instructions. A total of 1 µg of total RNA was transcribed into cDNA using the LunaScript RT supermix cDNA synthesis kit (New England Biolabs, Lpswich, MA, USA). For qRT-PCR, the EXPRESS SYBR Green ER reagent (Life Technologies, Inc., Carlsbad, CA, USA) was used in a real-time PCR system CFX96 from Bio-Rad. Cycling conditions used for all the qRT-PCRs were 3 min at 95 °C followed by 40 cycles of 15 s at 95 °C and 45 s at 60 °C, a common step for annealing and extension, at which data were collected. Rpl32 was used as an internal standard for normalization [8]. All experiments with NRVCMs were performed in hexaplicate and repeated three times.

### 4.8. Protein Preparation and Immunoblotting

For protein isolation, NRVCMs were lysed by two to three freeze-thaw cycles in RIPA lysis buffer containing 50mM Tris, 150mM NaCl, 1% Nonidet P-40, 0.5% sodium deoxycholate and 0.2% SDS, along with phosphatase inhibitor II, phosphatase inhibitor III and protease inhibitor mixture (Roche Applied Science). For protein harvest from mouse tissue or human hearts, a Precellys homogenizer with coarse and fine plastic beads (Peqlab, Germany) was employed. Cell debris in both methods was removed by centrifugation and protein concentration was determined photometrically by the DC assay method (Bio-Rad, Feldkirchen, Germany) against BSA serial dilutions. Protein samples were first resolved by 10% SDS-PAGE, before transferring to a nitrocellulose membrane and subsequently immunoblotted with the target-specific primary antibodies. The overnight application of mono- or poly-clonal primary antibodies was followed by incubation with a suitable HRP-coupled secondary antibody (1:10,000; Santa Cruz Biotechnology, Dallas, TX, USA) or fluorescent antibody Alexa Fluor 546 (for Tubulin only). Finally, protein band visualization was achieved using a chemiluminescence kit (GE Healthcare) and was detected on an imaging system (FluorChem Q; Biozym). A quantitative densitometry analysis was performed using the ImageJ version 1.46 software (National Institutes of Health) and plotted using Graphpad relative to control. All conditions were maintained in triplicates and repeated thrice.

### 4.9. Human Heart Samples

Left ventricular myocardial samples were taken from the explanted hearts of patients (NF = 5, HCM = 7) with the end-stage heart failure as characterized by the New York Heart Association, heart failure classification IV and thus undergoing heart transplantation. All procedures were performed in accordance with the ethical committee of the medical school of the University of Goettingen in Germany. The explanted hearts were acquired directly in the operation room during surgery and immediately placed in pre-cooled cardioplegic solution (in mmol/l: NaCl 110, KCl 16, MgCl_2_ 16, NaHCO_3_ 16, CaCl_2_ 1.2 and glucose 11). The samples for immunoblots were frozen in liquid nitrogen and stored at -80 degrees immediately after excision.

### 4.10. SRF Luciferase Assay

The SRF reporter gene assays shown in this study were performed on NRVCMs as described previously [8]. Briefly, cells were infected with several combinations of viruses expressing SH3BGR (50 ifu), miRSH3BGR (100 ifu) and RhoA (50 ifu), where LacZ and miRNeg served as controls or filler viruses to maintain an equal count of viruses, along with adenovirus Ad-SRF-RE-luciferase (20 ifu) carrying a firefly luciferase and Ad-Renilla-luciferase carrying (5 ifu) Renilla luciferase (for normalization of the measurements). SRF reporter gene assays were performed using a dual-luciferase reporter assay kit (Promega, Madison, WI, USA), according to the manufacturer’s guidelines. Chemiluminescence was measured photometrically on an Infinite M200 PRO system (Tecan, Life Science). All the experiments were performed in hexaplicate and repeated three times.

### 4.11. RhoA Inhibitor Usage

Clostridium botulinum-derived exoenzyme C3-transferase and its dominant-negative point mutant C3-E174Q (glutamate to glutamine at aa 174) were used to treat the cells for RhoA inhibition activity. Briefly, C3-E174Q mutant as control and C3-WT as treatment were used. NRVCMs were treated for 12 h at a concentration of 1ug/mL before harvesting.

### 4.12. Statistical Analysis

All results shown are the means ± S.E. unless stated otherwise. The statistical analyses of the data were performed using a two-tailed Student’s *t-*test for every experimental analysis. If necessary, two-way ANOVA (followed by Student–Newman–Keuls post hoc tests when appropriate) was applied. *p* values of less than 0.05 were considered statistically significant for all experiments.

## Supplementary Materials

The following are available online at https://www.mdpi.com/article/10.3390/ijms222011042/s1.

## Abbreviations

CHD	Congenital heart disease
DS	Down’s syndrome
SH3BGR	SH3-binding glutamic acid rich
Nppa	Natriuretic peptide A
Nppb	Natriuretic peptide B
NRVCM	Neonatal rat ventricular cardiomyocytes
RhoA	Ras Homolog family member A
SRF	Serum Response Factor
YAP	Yes associated protein
