# Supplementary material for: SH3-Binding Glutamic Acid Rich-Deficiency Augments Apoptosis in Neonatal Rat Cardiomyocytes
Source: Int J Mol Sci. 2021 Oct 13;22(20):11042. doi: 10.3390/ijms222011042 (PMC8541172; doi:10.3390/ijms222011042)
Supplement: Supplementary file 1 [file ijms-22-11042-s001.zip › ijms-1354790-supplementary.pdf]

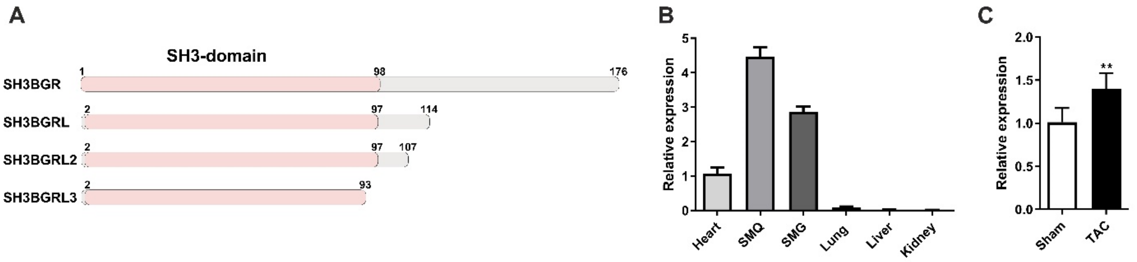

Supplementary figure S1- Expression levels of SH3BGR at transcript level- A) SH3BGR family proteins harboring conserved SH3 domain. B) Tissue distribution of SH3BGR at RNA level whereit is found to be expressed in mainly heart and skeletal muscle. C) Upon transverse aortic constriction in mice heart, SH3BGR levels were upregulated as compared to Sham operated mice at RNA level. Statistical calculations were carried out using two-tailed student’s t-test. **: $p<0.01$. SMQ: Skeletal muscle quadriceps, SMG: Skeletal muscle gracilis, TAC: Transverse aortic constriction.

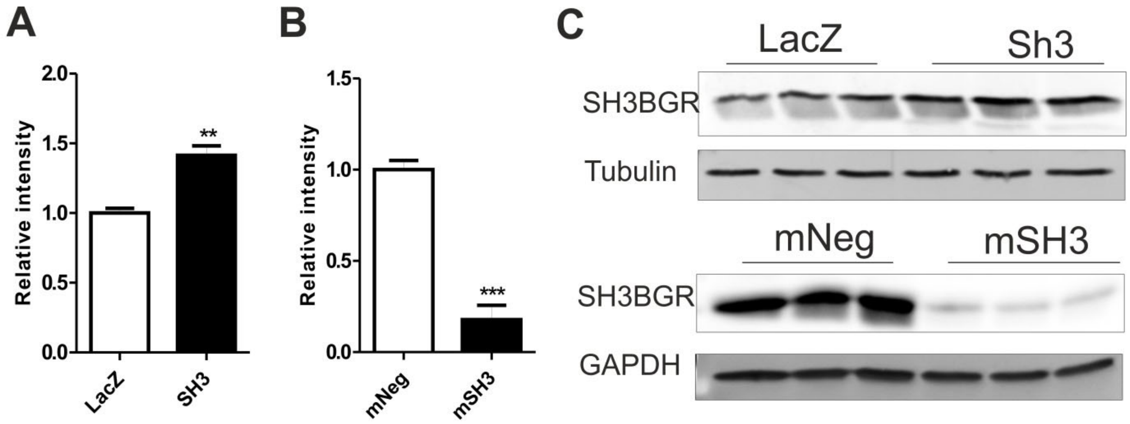

Supplementary figure S2- Overexpression and knockdown constructs of SH3BGR-Overexpression and knockdown of SH3BGR at portein level as compared to LacZ and miRNeg as the respective controls is shown in A-C. Statistical calculations were carried out using two-tailed student's t-test. **: p<0.01, ***: p<0.001.

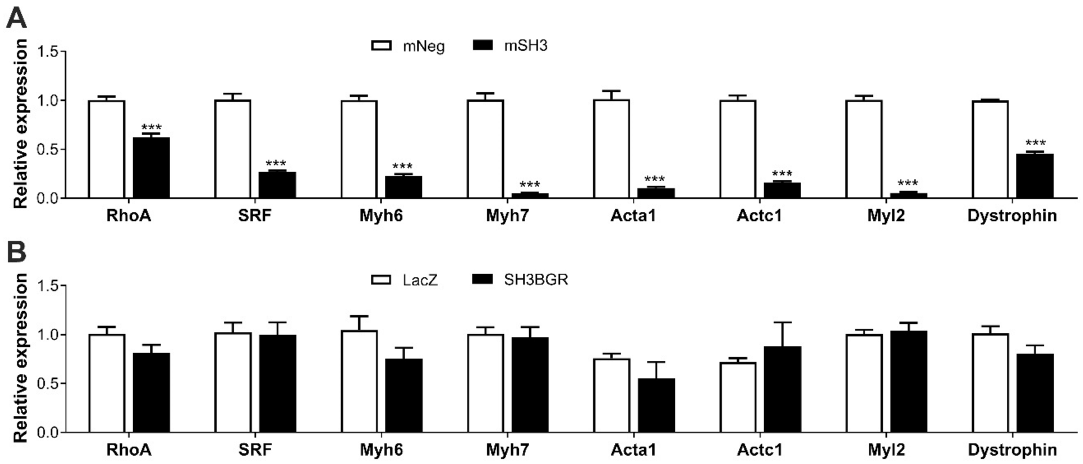

Supplementary figure S3- Effect of SH3BGR on SRF target genes- A) Knockdown of SH3BGR significantly reduced transcript levels of SRF target and sarcomeric protein. B) Overexpression of SH3BGR did not alter the downstream targets of SRF significantly. Statistical calculations were carried out using two-tailed student's t-test. ***: $p<0.001$. Myh6: myosin heavy chain 6, Myh7: myosin heavy chain 7, Myl2: myosin light chain2, Actc1: Actin Alpha Cardiac Muscle 1, Acta1: Actin Alpha 1, Skeletal Muscle.

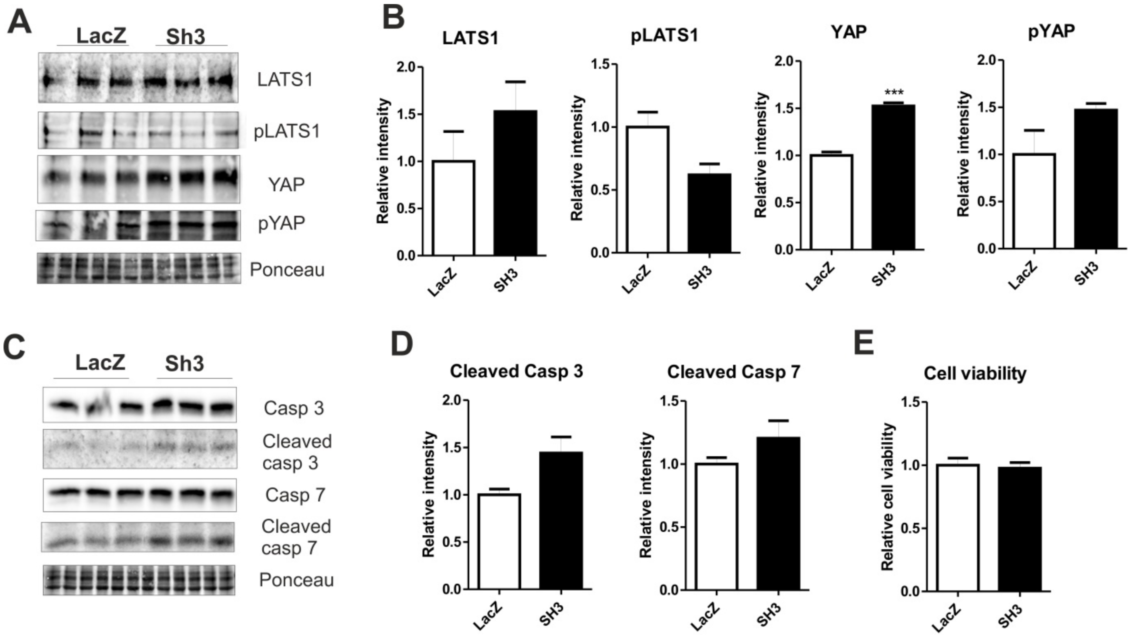

Supplementary figure S4- Effect of overexpression of SH3BGR on cellular apoptosis and Hippo signalling pathway- On overexpression of SH3BGR, we observed no significant change in the expression levels of LATS1 and its phosphorylated form pLATS1 after immunoblotting as observed in A and B. Although we could see upregulation of total YAP levels, there was no significant difference in the pYAP and overall, no effect on Hippo signalling upon overexpression. Furthermore, the expression levels of the cleaved fragment of executor caspases 3 and 7 responsible for apoptosis were also not significantly elevated as observed in C and D after immunoblotting indicating not much apoptosis taking place on SH3BGR overexpression which was also in line with the results of cell viability where we observed no significant effect on survival of NRVCMs as observed in E. Statistical calculations were carried out using two-tailed student's t-test. ***: $p<0.001$. LATS1: Large tumor suppressor kinase 1, pLATS1: phosphorylated LATS1, YAP: pYAP: phosphorylated YAP.nal Regulator,
